# Risk of Cardiovascular Disease in a Traditional African Population with a High Infectious Load: A Population-Based Study

**DOI:** 10.1371/journal.pone.0046855

**Published:** 2012-10-11

**Authors:** Jacob J. E. Koopman, David van Bodegom, J. Wouter Jukema, Rudi G. J. Westendorp

**Affiliations:** 1 Department of Gerontology and Geriatrics, Leiden University Medical Center, Leiden, The Netherlands; 2 Department of Gerontology and Geriatrics, Leiden University Medical Center, Leiden, The Netherlands; 3 Department of Cardiology, Leiden University Medical Center, Leiden, The Netherlands; 4 Department of Gerontology and Geriatrics, Leiden University Medical Center, Leiden, The Netherlands; 5 Leyden Academy on Vitality and Ageing, Leiden, The Netherlands; Innsbruck Medical University, Austria

## Abstract

**Background:**

To test the inflammatory origin of cardiovascular disease, as opposed to its origin in western lifestyle. Population-based assessment of the prevalences of cardiovascular risk factors and cardiovascular disease in an inflammation-prone African population, including electrocardiography and ankle-arm index measurement. Comparison with known prevalences in American and European societies.

**Methodology/Principal Findings:**

Traditional population in rural Ghana, characterised by adverse environmental conditions and a high infectious load. Population-based sample of 924 individuals aged 50 years and older. Median values for cardiovascular risk factors, including waist circumference, BMI, blood pressure, and markers of glucose and lipid metabolism and inflammation. Prevalence of myocardial infarction detected by electrocardiography and prevalence of peripheral arterial disease detected by ankle-arm index. When compared to western societies, we found the Ghanaians to have more proinflammatory profiles and less cardiovascular risk factors, including obesity, dysglycaemia, dyslipidaemia, and hypertension. Prevalences of cardiovascular disease were also lower. Definite myocardial infarction was present in 1.2% (95%CI: 0.6 to 2.4%). Peripheral arterial disease was present in 2.8% (95%CI: 1.9 to 4.1%).

**Conclusions/Significance:**

Taken together, our data indicate that for the pathogenesis of cardiovascular disease inflammatory processes alone do not suffice and additional factors, probably lifestyle-related, are mandatory.

## Introduction

The pathogenesis of cardiovascular disease has long been described as an accumulation of lipids in a dysfunctional endothelial wall, driven by lifestyle-related factors such as smoking, dyslipidaemia, dysglycaemia, obesity, and hypertension. Since the end of the millennium, it has been commonly accepted that inflammatory processes play a prominent role in atherosclerosis, reclassifying cardiovascular disease as a chronic inflammatory disorder [1]–[3]. Still, the exact balance between lifestyle and inflammation in its causality has remained unclear. There are both indications of subendothelial lipid retention [4] and inflammation [1] as being the primary causative factor.

The lifestyle-related risk factors are mainly present in western societies, which have experienced an epidemiologic and demographic transition. In these countries, cardiovascular disease is one of the major causes of morbidity and the most common cause of mortality. In developing countries, where these risk factors are absent, infectious diseases prevail [5]. Without the public health care of western societies, infectious diseases are highly lethal. Selectively those individuals that resist the high infectious pressure by a strong inflammatory response will survive, resulting in a population expressing a selectively proinflammatory immune system [6]–[10]. If cardiovascular disease is an inflammatory disease, it can be expected to be provoked by such a proinflammatory status. Indeed, with the progressing control of lethal infectious diseases, cardiovascular pathology is becoming more frequent in developing countries, especially in semideveloped environments where a western lifestyle concurs with a high infectious load [5], [11], [12]. Within western countries, the cardiovascular risk of migrants from developing countries remains elevated [12]–[14]. Inflammatory markers are used to predict cardiovascular risk [2], [3]. These figures reinforce the dominant role of inflammation in cardiovascular pathogenesis. However, the occurrence of cardiovascular disease in the remote and rural regions of developing countries, where a high infectious load induces proinflammatory phenotypes but a western lifestyle is absent, has infrequently been studied [15]–[17]. Knowledge on these data could further unravel the roles of lifestyle and inflammation as the origins of cardiovascular disease.

We have conducted a large population study in rural Ghana in West Africa. This study population has only recently started to experience an epidemiologic transition; the elderly are therefore not only a subgroup that has survived the pretransitional past, but have also been exposed to a pretransitional environment during the largest part of their lives [18]. Previous immunologic and genetic studies have shown this population to exhibit a proinflammatory immune status [8]–[10]. A previous study in a comparable population of forager-horticulturalists in Bolivia provided a first indication of a low prevalence of cardiovascular disease in populations where a western lifestyle is uncommon [7]. Our study population concerns a purely horticultural population where lifestyle-related risk factors are even more exceptional. This study firstly extends previous observations with a detailed assessment of cardiovascular health in a traditional African population, including electrocardiography and ultrasound measurement of ankle-arm indexes.

## Methods

### Research area

The Upper East Region in Ghana is remote, rural, and one of the least developed regions of the country. The vast majority of the inhabitants are involved in non-commercial agriculture performed by manual labor [19]. The yearly per capita income averages US$ 135 [20]; 88% of the households lives in poverty [21]. Infectious diseases are the main causes of death [22].

Since 2002, we have registered and followed a traditional horticultural population in the Garu-Tempane District in the Upper East Region occupying a research area of 375 km^2^ with approximately 25,000 inhabitants living in circa 40 villages. Families are polygamous and live as extended families in compounds. Migration is less than 1% per year. Hospital care is absent; the nearest physician is at 40 kilometers' distance. Vaccination of children has recently been introduced. Sewage disposal systems are non-existent.

Life expectancy at birth is 59 years. Based on changes in age-specific mortality rates over time, we have shown epidemiologic transition in this population to have started only recently with declines in mortality rates at young ages. Generations aged 50 years or more are unaffected by these changes and conform to a pretransitional pattern of survival [18].

All inhabitants have been registered and of each are known the name, age, sex, tribe, location of living. We determined the total fertility rate, defined as the median number of born children per postreproductive woman, based on fertility data gathered in the research area in 2003 [23]. For each household, we determined the household property value in 2007 according to the Demographic and Health Survey method [24]. In 2008, we determined the prevalences of infections by malaria species by PCR of blood samples and those by helminths and protozoa by PCR of stool samples [25]. A more elaborate description of this cohort has been given elsewhere [18], [23], [24]. This study was conducted within this cohort.

### Study sample

Based on our demographic cohort registration up to 2009, we targeted all inhabitants aged fifty years and older, counting 2,648 individuals. We consecutively visited the villages in our research area during two field visits in 2009 and 2010 to approach the targeted inhabitants. Inclusion was restricted by the duration of both field visits. To ensure maximal participation, we set up a mobile field work station in different villages and, if necessary, brought less mobile participants by car. We included 924 individuals, of whom 607 also underwent electrocardiographic measurement. Of the approached inhabitants, 4.4% could not participate due to death of the individual since the last registration, 3.2% refused participation, 2.8% was absent from the research area during our visit because of migration or travelling, and 4.2% did not participate for other reasons. At the station, the identity of the participant was confirmed, the personal data in the cohort registration was checked, and clinical and electrocardiographic investigations were performed. In addition, population-wide blood plasma samples of 266 individuals were available from 2008. On these, biochemical investigations were performed.

### Ethical approval

Ethical approval was given by the Ethical Review Committee of Ghana Health Services, the Committee Medical Ethics of the Leiden University Medical Center, and by the local chiefs and elders. Because of illiteracy, informed consent was obtained orally from the participants. A consent form was designed with an explanation on the purpose and conduction of this research project. This form was to be read out to each participant in his own language; consent was given verbally in the local language. Participation was only proceeded after verbal consent of the participant. The full text of the form was approved by the Ethical Review Committee of Ghana Health Services.

### Clinical investigations

Height and weight were measured by a calibrated length scale and weighing scale. Body mass index (BMI) was calculated as weight divided by squared height. Waist circumference was measured while standing by a measure tape at umbilical level. In a lying and resting position, blood pressure was measured at one arm using a calibrated sphygmomanometer and a Littman stethoscope. Hypertension was determined by these blood pressures and classified as stage I, stage II, or isolated systolic hypertension. Hypertension was defined as stage I for systolic blood pressures of 140 to 160 mmHg and/or diastolic blood pressures of 90 to 100 mmHg, as stage II for systolic blood pressures of 160 mmHg onward and/or diastolic blood pressures of 100 mmHg onward, and as isolated systolic hypertension for systolic blood pressures of 140 mmHg onward with diastolic blood pressures below 90 mmHg [26]. Systolic blood pressures were measured on the dorsalis pedis artery and the posterior tibial artery at both ankles using a calibrated sphygmomanometer and a doppler ultrasound machine (Imex DOPCT+). As a measure of peripheral arterial disease, the ankle-arm index was calculated by dividing the average systolic pressure of both arteries of each ankle by the systolic arm pressure. An index below 0.9 of either ankle indicates peripheral arterial disease [27]. Glucose whole blood concentration was measured on a capillary blood sample from a finger (Roche Accutrend Plus). Whole blood concentrations are circa 11% lower than plasma concentrations.

### Biochemical investigations

In 2008, venous blood samples were collected in the morning, carried on ice, centrifuged within four hours, after which plasma was kept frozen during storage and transportation at minimally −20°C [9]. Blood samples were analysed in our institution's Central Laboratory for Clinical Chemistry. Circulating plasma levels were determined for triglycerides, total cholesterol, apolipoprotein-B100 (apoB100), apolipoprotein-A1 (apoA1), C-reactive protein (CRP), and interleukin-6 (IL6). Measurements for triglycerides and total cholesterol were performed by colorimetric assays (Roche Modular P800); for apoB100, apoA1, and CRP by high-sensitivity immunoturbidimetric assays (Roche Cobas Integra); for IL6 by sandwich chemiluminescent immunoassay (Randox Evidence Investigator Biochip). All biochemical markers were measured in plasma; these concentrations are circa 3% lower than serum concentrations [27]. ApoB100 is associated with low-density lipoprotein (LDL) cholesterol, apoA1 with high-density lipoprotein (HDL) cholesterol. The apoB100 concentration and the ratio of the concentrations of apoB100 over apoA1 are strongly predictive risk factors of cardiovascular disease [27]. CRP and IL6 are proinflammatory markers.

### Criteria of cardiovascular risk factors

Cutoff values for cardiovascular risk factors were derived from the standardly used guidelines of the Adult Treatment Panel III [27] and the Joint National Committee on Prevention, Detection, Evaluation, and Treatment of High Blood Pressure [26]. Other guidelines were used for criteria not included herein, including the concentration of apoB100 and the ratio of the concentrations of apoB100 over apoA1 [28] and the concentration of CRP [29]. No commonly accepted cutoff value is available for IL6.

### Electrocardiographic investigations

A twelve-lead electrocardiogram (ECG) was recorded for twenty seconds in a lying and resting position (Schiller AT-104 PC). All ECGs were assessed by an experienced cardiologist according to the Minnesota criteria [30]. The cardiologist was blinded for the participants' characteristics other than age and sex. The ECGs were classified for indications of myocardial infarction, being absent, possibly or definitively present, and for myocardial ischaemia-like changes, being absent, minor, or major. For both, the localisations were classified, being anterior, septal, inferior, and/or lateral. Merely anteriorly localised ischaemia-like changes were excluded from analysis (6.4%), as these are reported to be little specific in African ethnicities [31]–[33]. Indications of definite infarction included major and moderate pathological Q waves and QS patterns (codes 1-1 and 1-2). Indications of possible infarction included minor pathological Q waves and QS patterns (code 1-3). Definite ischaemia-like changes included major ST depression, T wave inversion, and ST elevation (codes 4-1, 4-2, 5-1, 5-2, and 9-2). Minor ischaemia-like changes included minor ST depression and T wave inversion and complete left bundle branch block (codes 4-3, 4-4, 5-3, 5-4, and 7-1).

### Reference populations

We compared the findings from our traditional African study population with those of western societies. As reference populations, we used three population-based studies in the United States and Europe for individuals at ages from 45 or 50 years. Data on the general population in the United States were supplied by the National Health and Nutrition Examination Survey (NHANES) for the prevalences of electrocardiographically measured coronary arterial disease (*n* = 6,201, median age 63 years, 45.3% males) [34], the prevalence of peripheral arterial disease measured by ankle-arm index (*n* = 1,868, median age 61 years, 48.4% males), the prevalence of hypertension (*n* = 2,135, median age 62 years, 46.6% males), and the distributions of cardiovascular risk factors and inflammatory markers (*n* = 2,323, median age 62 years, 46.1% males) [35]. Data on the general European population were provided by a study in Belgium for the prevalences of electrocardiographically measured coronary arterial disease (*n* = 29,419, median age 52 years, 77.7% males) [36] and by the CoLaus Study in Switzerland for the prevalence of hypertension and the distributions of cardiovascular risk factors and inflammatory markers (*n* = 3,515, median age 61 years, 45.4% males) [37], [38]. In the cases of Belgian prevalences of electrocardiographic abnormalities and the Swiss distribution of IL6, only sex-split data were available. For comparability, both were graphically displayed as averages weighed by the number of individuals per sex. For the reference populations, within the definition of hypertension was included the use of antihypertensive medication, within the definition of diabetes was included the use of antidiabetic medication, and within the definition of elevated levels of total cholesterol or LDL cholesterol was included the use of antidyslipidaemic medication. Otherwise, applied definitions and cutoff values were identical as described for our study.

### Analyses

Confidence intervals were calculated by Wilson's formula for prevalences and using the binomial distribution for medians. Individual associations of presence of cardiovascular disease with cardiovascular risk factors, inflammation, and socio-economic status were tested by logistic regression analysis, including age and sex as dependent variables. Statistical analyses were performed in PASW Statistics 18.

## Results

### General characteristics

Table 1 provides a description of the demographic, socioeconomic, and infectious characteristics of the study sample that participated in the electrocardiographic investigations. The overrepresentation of women and a heightened socioeconomic status compared to the total cohort population are results of the selection on age. Otherwise, the baseline characteristics of the study sample that participated in the electrocardiographic investigations were similar to those of the study samples that participated in the clinical and biochemical investigations as well as to those throughout the total cohort population.

**Table 1:** General characteristics of the Ghanaian study sample (age≥50 years).

Individuals *n*	610	
Households *n*	422	
Age *median (iqr) years*	66	(56–74)
Male sex *n* (*%*)	169	(27.5)
Total fertility rate *median (iqr) children per woman*	7	(6–9)
Household property value *median (iqr) US$*	1,050	(500–1,936)
Individuals infected *%*		
by malaria species	0077.5%	
by helminths	0021.4%	
by protozoa	0100.0%	

A description of the general characteristics is given for the study sample aged 50 years and older that participated in the electrocardiographic investigations. Total fertility rate is expressed as the number of born children per woman aged 45 years or more. Iqr – interquartile range.

### Cardiovascular risk factors

Table 2 summarises the clinical and biochemical investigations for the detection of cardiovascular risk factors concerning body size, blood pressure, glucose metabolism, lipid metabolism, and inflammation. Hypertension was classified as stage I, stage II, or isolated systolic hypertension. Plasma glucose concentrations were used to detect elevated cardiovascular risk from 5.6 mmol/l onward and to detect diabetes mellitus from 7.0 mmol/l onward [27]. The balance between HDL and LDL cholesterol was represented by the ratio of the plasma concentrations of apoB100 over apoA1. As markers of the inflammatory status, plasma concentrations of CRP and IL6, both proinflammatory cytokines, were used. In our study population, median levels of cardiovascular risk factors were low and prevalences of obesity, hypertension, dysglycaemia or diabetes, and dyslipidaemia were low. The prevalence of moderately elevated levels of CRP was low.

**Table 2:** Cardiovascular risk factors in the Ghanaian study sample (age≥50 years).

	Males		Females	
**Clinical investigations**	(*n* = 480)		(*n* = 444)	
Waist circumference *cm*	77	(73–81)	76	(72–80)
*≥102 (♂) or ≥88 cm (♀)*	0.0%	(0.0–0.8)	7.4%	(5.3–10.3)
Body mass index *kg/m^2^*	18.1	(16.5–19.4)	18.1	(16.6–19.7)
*≥25.0 kg/m^2^*	0.2%	(0.0–1.2)	1.4%	(0.6–2.9)
Diastolic blood pressure *mmHg*	75	(70–80)	70	(65–75)
Systolic blood pressure *mmHg*	120	(110–135)	120	(110–135)
Hypertension				
stage I	16.7%	(13.6–20.3)	13.7%	(10.8–17.3)
stage II	9.0%	(6.7–11.9)	8.8%	(6.5–11.8)
isolated systolic	15.7%	(12.7–19.2)	16.2%	(13.1–19.9)
Glucose *mmol/l*	3.8	(3.3–4.4)	4.0	(3.6–4.5)
*≥5.6 mmol/l*	6.0%	(4.3–8.6)	6.3%	(4.5–9.1)
*≥7.0 mmol/l*	1.0%	(0.4–2.4)	1.4%	(0.6–3.0)
**Biochemical investigations**	(*n* = 94)		(*n* = 172)	
Triglycerides *mmol/l*	0.80	(0.68–0.95)	0.95	(0.76–1.21)
*≥1.7 mmol/l*	4.3%	(1.7–10.4)	6.4%	(3.6–11.1)
Total cholesterol *mmol/l*	3.04	(2.49–3.51)	3.24	(2.81–3.74)
*≥5.2 mmol/l*	1.1%	(0.2–5.8)	1.7%	(0.6–5.0)
ApoB100 *g/l*	0.54	(0.47–0.64)	0.58	(0.49–0.69)
*≥0.9 g/l*	2.1%	(0.6–7.4)	2.9%	(1.3–6.7)
Ratio of apoB100 over apoA1	0.58	(0.45–0.68)	0.52	(0.43–0.66)
*≥0.9 (♂) or ≥0.8 (♀)*	5.3%	(2.3–11.9)	12.8%	(8.7–18.7)
C-reactive protein *mg/l*	1.06	(0.34–3.77)	0.95	(0.44–2.35)
*3.0–10.0 mg/l*	19.1%	(12.5–28.3)	11.6%	(7.7–17.3)
Interleukin-6 *ng/l*	2.28	(1.47–3.23)	1.84	(1.37–2.42)

Distributions are given as medians (interquartile ranges), prevalences are given as percentages (95% confidence intervals) meeting the indicated risk factor criteria, all for those aged 50 years and older. Hypertension has been classified as stage I for diastolic blood pressures of 90 to 100 mmHg and/or systolic blood pressures of 140 to 160 mmHg, as stage II for diastolic blood pressures from 100 mmHg onward and/or systolic blood pressures from 160 mmHg onward, and as isolated systolic for systolic blood pressures from 140 mmHg onward with diastolic blood pressures lower than 90 mmHg. ApoA1 – apolipoprotein-A1. ApoB100 – apolipoprotein-B100.

**Figure 1:**
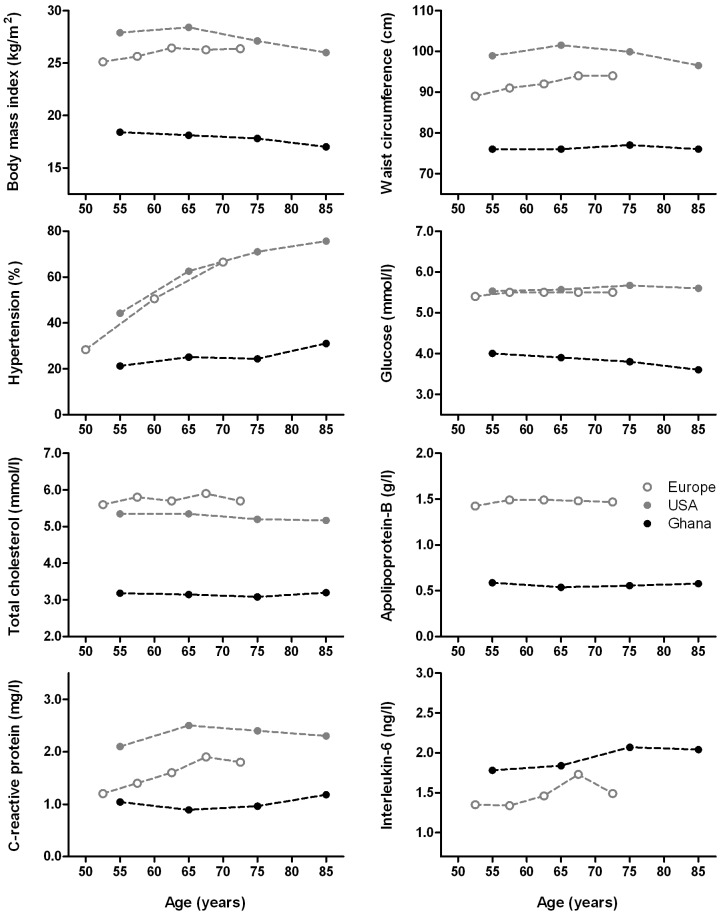
Cardiovascular risk factors in the Ghanaian study sample compared with the American and European reference populations over age. Distributions over age are given as medians. Prevalences over age of hypertension are given as percentages, including stage I, stage II, and isolated systolic hypertension. Values for age represent midpoints of age intervals, because of different age groups used for the reference populations.

In Figure 1, the distributions of cardiovascular risk factors in our study population are compared to those in the western reference populations. Over the age of 50 years, in Ghana lower median values or prevalences were found for all cardiovascular risk factors describing body size, blood pressure, and glucose and lipid metabolism compared to the United States and Europe. For the inflammatory markers, median levels of CRP were lower, but levels of IL6 were higher.

### Cardiovascular disease

Table 3 shows the prevalences of cardiovascular disease in our study population. We used electrocardiography to detect coronary arterial disease, classified as possible or definite myocardial infarction and minor or definite myocardial ischaemia-like changes. Prevalences of myocardial infarction and myocardial ischaemia-like changes were low. We used measurement of ankle-arm index to detect peripheral arterial disease, defined as an ankle-arm index below 0.9 of either leg. The prevalence of peripheral arterial disease was low; median ankle-arm indexes were high.

**Table 3:** Cardiovascular disease in the Ghanaian study sample (age≥50 years).

	Males		Females	
**Electrocardiographic investigations**	(*n* = 168)		(*n* = 439)	
Myocardial infarction				
definite	1.8%	(0.6–5.1)	0.9%	(0.4–2.3)
possible	4.2%	(2.0–8.4)	2.1%	(1.1–3.8)
Myocardial ischaemia-like changes				
definite	13.7%	(9.3–19.7)	10.0%	(7.6–13.2)
minor	2.4%	(0.9–6.0)	3.2%	(1.9–4.6)
**Clinical investigations**	(*n* = 480)		(*n* = 444)	
Ankle-arm index	1.16	(1.10–1.24)	1.14	(1.06–1.22)
Peripheral arterial disease	2.3%	(1.3–4.1)	3.4%	(2.1–5.5)

Prevalences are given as percentages (95% confidence intervals) and distributions are given as medians (interquartile ranges), all for those aged 50 years and older. Peripheral arterial disease has been defined as an ankle-arm index below 0.9.

**Figure 2:**
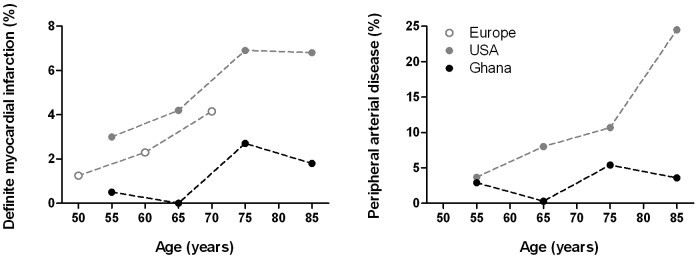
Cardiovascular disease in the Ghanaian study sample compared with the American and European reference populations over age. Prevalences over age are given as percentages. Peripheral arterial disease has been defined as an ankle-arm index below 0.9. Definite myocardial infarction has been detected by electrocardiography. Values for age represent midpoints of age intervals, because of different age groups used for the reference populations.

As shown in Figure 2, the prevalences of both coronary and peripheral arterial disease in Ghana were lower compared to the western reference populations, using identical clinical and electrocardiographic criteria. A similar pattern was found for myocardial ischaemia-like changes. Moreover, Ghanaian prevalences of coronary and peripheral arterial disease increased less over age.

### Individual associations

For the individuals affected by cardiovascular disease, no associations could be found with cardiovascular risk factors, inflammation, or socioeconomic status.

## Discussion

This study aimed to determine cardiovascular health in a traditional population in Ghana in West Africa after the age of 50 years. Our study population is demographically and economically characterised by an underdeveloped pretransitory status relative to western countries. In this adverse environment, individuals are exposed to a high infectious load, favouring survival of those with a proinflammatory immune status. Compared to American and European societies, we indeed found common proinflammatory profiles, but low prevalences of cardiovascular risk factors associated with a western lifestyle, including obesity, hypertension, dysglycaemia or diabetes, and dyslipidaemia. In this population, prevalences of coronary and peripheral arterial disease measured by electrocardiography and ankle-arm index were correspondingly low.

For some cardiovascular risk factors no formal determination was possible, including smoking behaviour, alcohol use, diet composition, and lack of physical exercise. Tobacco is self-grown and beer is self-brewed. The availability of both is dependent on the harvest, as commercial distribution of cigarettes and alcoholic beverages is locally absent. Their regular use is more or less a social taboo. Based on our long-lasting experiences, regular smoking and drinking among men is common, estimated to concern one third and two thirds of the population, respectively, but the quantities are likely to be limited when compared to developed countries. Women do not publicly smoke or drink, but we presume women to exhibit these behaviours privately in smaller proportions. Physical inactivity is exceptional and western diet is absent. It should be noted that most inhabitants in the research area provide for their own living by non-commercial agriculture performed by manual labour. The scarcity of electricity and machinery necessitates physical exercise for farming and housekeeping. Transportation is almost always by foot or bicycle. The savannah climate is dry with only one rainy season of variable duration, hindering agriculture. The limited variety of crops that can be grown in this environment – e.g. millet, corn, okra, beans, onions, and tomatoes – and the omnipresent low social economic standard restrict the ingestion of fat and glucose. Commercial distribution of conserved food has remained financially unattainable for most.

Although the prevalence of cardiovascular disease was low, cardiovascular disease was not absent in our study population. This indicates that low prevalences of obesity, dysglycaemia or diabetes, and dyslipidaemia do not entirely prevent the occurrence of cardiovascular disease. The presence of hypertension is relatively uncommon, but still present in about one fifth of the population. It may be relatively important for cardiovascular pathogenesis in this population. Moreover, some do smoke and drink, but the consumption and attribution are difficult to estimate. Other factors independent of lifestyle, such as genotype, may also render individuals susceptible. In line with this, for those few who are affected, we have been unable to find any association with the cardiovascular risk factors as measured in this study. In this study, genotypes have not been assessed. Recently, we have collected mouth swabs in our study population for genetic analyses in future research.

Previous research on risk factors of cardiovascular disease has shown that in Africa, equal to western societies, cardiovascular disease can be mainly attributed to obesity, dysglycaemia, dyslipidaemia, smoking, and hypertension [11]–[13]. Pathogenetic mechanisms have been described by which lifestyle-related risk factors drive cardiovascular disease [4], [40]. However, these studies are based on urban regions with westernised risk profiles. Data from populations without a western lifestyle, as our study population, are needed to confirm that these lifestyle-related risk factors are primarily responsible for cardiovascular disease to develop.

In our study population, all individuals were infected by protozoa, while infections by helminths and malaria species were highly endemic. This high infectious load, together with little public and personal hygiene and little medical care, renders infectious diseases as the main cause of death. Previously, we have shown that selectively those individuals that express a proinflammatory immune system survive up to older ages [8]–[10]. This selection of a proinflammatory status is confirmed by the higher levels of the proinflammatory cytokine interleukin-6 (IL6) in our study population compared to western societies. Different from IL6, we found lower levels of the inflammatory marker C-reactive protein (CRP) in our study population compared to western societies. Plasma levels of CRP are highly elevated during acute infections. During chronic, subclinical infections, CRP levels may not be elevated. In populations endemic for infections by malaria and helminth species, these levels can be rather within the normal range [41]–[43]. Probably, in these instances, a systemic inflammatory response is not elicited, as the immune system has become effectively able to restrain the pathogens or has become tolerant. Distinctive to chronic infections, however, chronic non-infectious inflammatory disorders are associated with moderately elevated levels of CRP [29]. Likely, instead of being a risk factor, these moderately elevated levels are an effect of the pathophysiological inflammatory processes and thus a marker of cardiovascular disease [44]–[46]. In line with this, we found less moderately elevated levels in our study population compared to western societies. Similar results have been obtained in other developing populations with high rates of infectious diseases in Southern America [47], [48]. The comparisons of CRP and IL6 levels with those in western societies are consistent when using other reports from western societies [49], [50].

Although we show that inflammation alone is not sufficient to cause cardiovascular disease, inflammation still plays a major role in its pathogenesis. This is reflected by the high prevalences of cardiovascular disease in regions where a western lifestyle coincides with a high infectious load [5], [11]–[13]. In addition, migrants from developing regions are affected by cardiovascular disease in western societies at younger ages and with higher mortality [11]–[14]. These populations are likely to have developed a proinflammatory immune status and are thereby probably more susceptible to develop inflammatory disease, such as cardiovascular disease. Even in developed countries, such predisposing processes can be effectuated through adverse environmental conditions [51], [52]. These notions are supported by the observation of higher levels of CRP in African Americans compared to white Americans [50], [53] and the beneficial effects of antidyslipidaemic therapy in individuals with elevated CRP [54].

This study has several limitations. First, for the registration of glucose and lipid parameters, blood samples have been taken in the morning. Although breakfast is uncommon, participants have not been requested to fast. Observed glucose and triglyceride levels could have been overestimated in this population and fasted values could be even lower than reported here. This reinforces the present interpretation of the study. Second, we have not been able to cover all cardiovascular risk factors, leaving smoking, alcohol use, diet composition, physical exercise, and genetic factors. Third, because of selection on age, women are overrepresented, demanding some prudence in the interpretation of the data for men.

Electrocardiographic criteria are reliable for the distinction of myocardial infarction. However, myocardial ischaemia-like changes are less distinctive [36]. Apart from coronary arterial disease, other disorders can effectuate these changes in repolarisation [33]. Moreover, in African ethnicities, these changes in repolarisation are little specific for coronary arterial disease [31], [32], [55]. Therefore, true prevalences of cardiovascular disease might be even lower than reported in this study. Unknown as well remain possible confounding selective processes. In our study population, the mortality due to cardiovascular disease is undetermined. If high, those affected could have deceased before being detected. When a selection exists for physically fit individuals to survive, those prone to develop cardiovascular disease might not live to older ages. However, these suppositions are contradicted by our observation of the few cases that were found with a slightly increasing trend over age.

This study determines the population-wide prevalences of peripheral and coronary arterial disease in a traditional African population by measurement of ankle-arm index and electrocardiography. These prevalences have not been described earlier for developing populations without a western lifestyle in relation to lifestyle and inflammation. First, this knowledge is of importance for underdeveloped populations themselves to guide preventive and curative policies. Worldwide, the prevalence of cardiovascular disease is rapidly increasing, especially in developing regions parallel to urbanisation and westernisation [11], [12]. As we show, also in these regions, it can be worthwhile to emphasise on the research and prevention of lifestyle-related risk factors for the control of cardiovascular disease. Second, this knowledge is of equal importance of western societies. Of today's African Americans, a large majority is genetically related to traditional populations in West Africa [39]. Predominantly these and other migrant populations suffer from a burden of cardiovascular disease, even after lifelong residence in western society [11]–[14]. It remains unclear to what extent this disparity can be explained by biological, socioeconomic, and cultural differences. Possibly, these ethnicities have been programmed through selection or through development to be more proinflammatory. Nonetheless, our study confirms that preventive and curative policies may be expected to be successful when addressing particular aspects of lifestyle.

In conclusion, we have shown that in a traditional non-western African population, despite promotion of inflammation, cardiovascular disease infrequently develops because of low prevalences of lifestyle-related cardiovascular risk factors compared to American and European societies. Together, these results indicate that cardiovascular disease is not primarily caused by inflammatory processes, but a western lifestyle is a prerequisite for its development. This provides even further arguments to prioritise the promotion of a healthy lifestyle, both in developed and in developing countries.
